# Supplementary material for: Genetic risk in extremely early onset type 1 diabetes
Source: medRxiv. 2025 Dec 19:2025.12.18.25342362. Preprint. [Version 1] doi: 10.64898/2025.12.18.25342362 (PMC12723774; doi:10.64898/2025.12.18.25342362)
Supplement: Supplement 1 [file media-1.pdf]

EXE-T1D Consortium members:
Department of Clinical & Biomedical Sciences, University of Exeter Medical School, Exeter, UK
Rebecca A Dobbs, Andrew T Hattersley, Michelle Hudson, Timothy J McDonald, Noel G Morgan, Kathryn Murrall, Sarah J Richardson, Suraj Ramchand, Dill Patel

Department of Immunobiology, School of Immunology & Microbial Sciences (SIMS), King's College London, UK
Paul Blair,

Center for Interventional Immunology, Benaroya Research Institute, Seattle, WA
Bradford Dimos, Cate Speake, Megan E Smithmyer

Translational Health Sciences, Bristol Medical School, University of Bristol, Southmead Hospital, Bristol, UK
Kathleen M Gillespie

Diabetes and Inflammation Laboratory, Centre for Human Genetics, Nuffield Department of Medicine, Bukhman Centre for Research Excellence in Type 1 Diabetes, Oxford NIHR Biomedical Research Centre, University of Oxford, Oxford, UK
Rachel E J Besser

Department of Paediatric Endocrinology and Diabetes, Oxford University Hospitals NHS Foundation Trust, Oxford, UK.
Rachel E J Besser

Department of Paediatric Endocrinology and Diabetes, Birmingham Women's and Children's NHS Foundation Trust, Birmingham, UK
Renuka P Dias

Mendy Korer - Participant Parent Representative
