# Supplementary material for: Genetic risk in extremely early onset type 1 diabetes
Source: medRxiv. 2025 Dec 19:2025.12.18.25342362. Preprint. [Version 1] doi: 10.64898/2025.12.18.25342362 (PMC12723774; doi:10.64898/2025.12.18.25342362)
Supplement: Supplement 2 [file media-2.pdf]

Supplementary Table 1. Cohort characteristics.

Cohort	Total (n)	Total T1D (n)	Total Controls (n)	Mean T1D onset (years) (95% CI)	<2y T1D onset (n)	2y-7y T1D onset (n)	7y-13y T1D onset (n)	13y+ T1D onset (n)	% Females T1D	% Females Controls	% European ancestry
**EXET1D/ EXTEND/ PRB**	10,696	293	10,403	2.3 (1.7–2.9)	242	33	10	8	44	50.5	97
**T1DGC**	16,076	6480	9596	7.8 (7.7–7.8)	352	2208	3084	836	49.3	51.5	100
**UKB**	367,318	NA	367,318	NA	NA	NA	NA	NA	NA	54.5	100
