# Supplementary material for: Genetic risk in extremely early onset type 1 diabetes
Source: medRxiv. 2025 Dec 19:2025.12.18.25342362. Preprint. [Version 1] doi: 10.64898/2025.12.18.25342362 (PMC12723774; doi:10.64898/2025.12.18.25342362)
Supplement: Supplement 3 [file media-3.pdf]

Supplementary Table 2. Classification of HLA haplotypes and associated abbreviation.

DR-DQ haplotype	Abbreviation
DRB1*07:01-DQA1*02-DQB1*02:02	DQ2.2
DRB1*03:01-DQA1*05-DQB1*02:01	DR3-DQ2
DRB1*08:01-DQA1*04-DQB1*04:02	DQ4.2
DRB1*01:0X-DQA1*01-DQB1*05:01	DQ5.1
DRB1*16:0X-DQA1*01-DQB1*05:02	DQ5.2
DRB1*14:01-DQA1*01-DQB1*05:03	DQ5.3
DRB1*15:02-DQA1*01-DQB1*06:01	DQ6.1
DRB1*15:01-DQA1*01-DQB1*06:02	DQ6.2
DRB1*13:01-DQA1*01-DQB1*06:03	DQ6.3
DRB1*13:02-DQA1*01-DQB1*06:04	DQ6.4
DRB1*13:02-DQA1*01-DQB1*06:09	DQ6.9
DRB1*11:0X-DQA1*05-DQB1*03:01	DQ7.5
DRB1*08:03-DQA1*06-DQB1*03:01	DQ7.6
DRB1*04:0X-DQA1*03-DQB1*03:02	DR4-DQ8
DRB1*07:01-DQA1*02-DQB1*03:03	DQ9.2
DRB1*09:01-DQA1*03-DQB1*03:03	DQ9.3
DRB1*04:0X-DQA1*03-DQB1*03:01	DQ7.3
