# Supplementary material for: Genetic risk in extremely early onset type 1 diabetes
Source: medRxiv. 2025 Dec 19:2025.12.18.25342362. Preprint. [Version 1] doi: 10.64898/2025.12.18.25342362 (PMC12723774; doi:10.64898/2025.12.18.25342362)
Supplement: Supplement 4 [file media-4.pdf]

Supplementary Table 3. Effect sizes of HLA variants from <7 year T1D GWAS and >13 year T1D GWAS and P-value from heterogeneity test.

Locus	<7 year effect size (ß)	>13 year effect size (ß)	*P*-value
DQ6.3	-2.42	-1.05	1.50E-02
*A*29:02*	-0.38	-0.04	4.30E-02
*B*18:01*	-0.59	-0.02	4.00E-02
*B*45:01*	-0.77	0.15	1.80E-02
DQ6.9	-1.67	-0.8	3.70E-02
*DPB1*15:01*	0.51	0.32	3.90E-03
RBM17	-0.64	-0.49	4.60E-02
DQ4.2	0.31	0.03	4.50E-02
*B*39:06*	1.99	0.89	6.50E-03
BNTNL2-DRA1	0.56	0.53	2.10E-02
*B*57:01*	0.63	0.48	4.70E-02
DRA1-DRB1	1.75	1.11	5.70E-03
DQ9.3	0.18	0.16	2.60E-02
ITGB7	0.22	0.41	4.70E-02
*A*02:05*	0.55	0.63	9.30E-03
