# Supplementary material for: Genetic risk in extremely early onset type 1 diabetes
Source: medRxiv. 2025 Dec 19:2025.12.18.25342362. Preprint. [Version 1] doi: 10.64898/2025.12.18.25342362 (PMC12723774; doi:10.64898/2025.12.18.25342362)
Supplement: Supplement 5 [file media-5.pdf]

Supplementary Table 4. Regression output comparing HLA contribution to <2 years type 1 diabetes onset.

Allele	Phenotype	OR	SE	Z	*P*-value
HLA A*24:02	Control	0.903	0.017	-6.122	9.25E-10
HLA A*24:02	2-7	0.993	0.018	-0.381	0.703
HLA A*24:02	7-13	0.957	0.018	-2.469	0.0136
HLA A*24:02	13+	0.883	0.021	-5.844	5.1E-09
HLA A*24:02	Control	0.903	0.017	-6.122	9.25E-10
HLA A*24:02	2-7	0.993	0.018	-0.381	0.703
HLA A*24:02	7-13	0.957	0.018	-2.469	0.0136
HLA A*24:02	13+	0.883	0.021	-5.844	5.1E-09
DR3	Control	0.950	0.018	-2.815	0.00487
DR3	2-7	0.970	0.020	-1.548	0.122
DR3	7-13	0.962	0.019	-2.006	0.0448
DR3	13+	0.967	0.023	-1.474	0.14
DR4	Control	0.966	0.017	-2.044	0.041
DR4	2-7	1.092	0.019	4.635	3.57E-06
DR4	7-13	1.109	0.018	5.601	2.13E-08
DR4	13+	1.145	0.022	6.208	5.38E-10
DR3/DR4	Control	0.658	0.012	-33.593	<2.2E-16
DR3/DR4	2-7	0.910	0.014	-6.865	6.63E-12

DR3/DR4	7-13	0.870	0.013	-10.386	<2.2E-16
DR3/DR4	13+	0.829	0.016	-11.817	<2.2E-16
DRB1*15:01-DQA1*01:02-DQB1*06:02	Control	1.279	0.017	14.406	<2.2E-16
DRB1*15:01-DQA1*01:02-DQB1*06:02	2-7	0.989	0.019	-0.580	0.562
DRB1*15:01-DQA1*01:02-DQB1*06:02	7-13	0.991	0.018	-0.466	0.641
DRB1*15:01-DQA1*01:02-DQB1*06:02	13+	0.996	0.022	-0.176	0.861
DRB1*07:01-DQA1*02:01-DQB1*03:03	Control	1.086	0.010	8.442	<2.2E-16
DRB1*07:01-DQA1*02:01-DQB1*03:03	2-7	1.009	0.011	0.838	0.402
DRB1*07:01-DQA1*02:01-DQB1*03:03	7-13	1.011	0.011	0.999	0.318
DRB1*07:01-DQA1*02:01-DQB1*03:03	13+	1.017	0.013	1.350	0.177
DRB1*13:02-DQA1*01:02-DQB1*06:09	Control	1.017	0.005	3.230	0.00124
DRB1*13:02-DQA1*01:02-DQB1*06:09	2-7	0.998	0.006	-0.300	0.765
DRB1*13:02-DQA1*01:02-DQB1*06:09	7-13	0.998	0.006	-0.354	0.723

DRB1*13:02-DQA1*01:02-DQB1*06:09	13+	1.004	0.007	0.645	0.519
DRB1*13:01-DQA1*01:03-DQB1*06:03	Control	1.081	0.011	6.872	6.31E-12
DRB1*13:01-DQA1*01:03-DQB1*06:03	2-7	1.007	0.013	0.583	0.56
DRB1*13:01-DQA1*01:03-DQB1*06:03	7-13	1.012	0.012	0.954	0.34
DRB1*13:01-DQA1*01:03-DQB1*06:03	13+	1.013	0.014	0.915	0.36
DRB1*08:01-DQA1*04:01-DQB1*04:02	Control	0.988	0.008	-1.405	0.16
DRB1*08:01-DQA1*04:01-DQB1*04:02	2-7	1.002	0.009	0.222	0.825
DRB1*08:01-DQA1*04:01-DQB1*04:02	7-13	0.993	0.009	-0.817	0.414
DRB1*08:01-DQA1*04:01-DQB1*04:02	13+	0.992	0.011	-0.704	0.481
HLA A*02:01	Control	0.953	0.027	-1.777	0.0756
HLA A*02:01	2-7	1.023	0.030	0.764	0.445
HLA A*02:01	7-13	1.025	0.029	0.852	0.394
HLA A*02:01	13+	1.028	0.035	0.789	0.43
HLA A*02:05	Control	0.990	0.006	-1.731	0.0834
HLA A*02:05	2-7	1.000	0.007	-0.004	0.996

HLA A*02:05	7-13	1.000	0.007	-0.019	0.985
A*02:05	13+	0.989	0.008	-1.416	0.157
HLA A*11:01	Control	1.055	0.013	4.007	6.15E-05
HLA A*11:01	2-7	1.002	0.015	0.109	0.913
HLA A*11:01	7-13	1.016	0.014	1.080	0.28
HLA A*11:01	13+	1.003	0.017	0.163	0.87
HLA B*18:01	Control	0.945	0.013	-4.555	5.24E-06
HLA B*18:01	2-7	1.006	0.014	0.402	0.688
HLA B*18:01	7-13	1.006	0.013	0.413	0.68
HLA B*18:01	13+	0.983	0.016	-1.082	0.279
HLA B*39:06	Control	0.911	0.007	-13.374	<2.2E-16
HLA B*39:06	2-7	0.974	0.008	-3.334	0.000855
HLA B*39:06	7-13	0.945	0.008	-7.531	5.04E-14
HLA B*39:06	13+	0.928	0.009	-8.306	<2.2E-16
HLA B*44:03	Control	1.078	0.013	5.826	5.68E-09
HLA B*44:03	2-7	1.011	0.014	0.732	0.464
HLA B*44:03	7-13	1.009	0.014	0.652	0.514
HLA B*44:03	13+	1.025	0.017	1.468	0.142
HLA B*45:01	Control	1.001	0.005	0.239	0.811
HLA B*45:01	2-7	0.992	0.005	-1.497	0.134

HLA B*45:01	7-13	0.996	0.005	-0.823	0.411
HLA B*45:01	13+	1.004	0.006	0.593	0.553
HLA DPB1*03:01	Control	0.906	0.019	-5.177	2.26E-07
HLA DPB1*03:01	2-7	1.001	0.021	0.054	0.957
HLA DPB1*03:01	7-13	0.983	0.021	-0.847	0.397
HLA DPB1*03:01	13+	0.958	0.024	-1.747	0.0806
HLA DPB1*04:02	Control	1.086	0.018	4.718	2.38E-06
HLA DPB1*04:02	2-7	0.984	0.019	-0.848	0.397
HLA DPB1*04:02	7-13	0.984	0.019	-0.871	0.384
HLA DPB1*04:02	13+	1.004	0.022	0.183	0.855
HLA DPB1*15:01	Control	0.999	0.005	-0.254	0.8
HLA DPB1*15:01	2-7	1.009	0.006	1.541	0.123
HLA DPB1*15:01	7-13	1.009	0.006	1.563	0.118
HLA DPB1*15:01	13+	1.007	0.007	0.978	0.328
HLA A*24:02	Control	0.903	0.017	-6.122	9.25E-10
HLA A*24:02	2-7	0.993	0.018	-0.381	0.703
HLA A*24:02	7-13	0.957	0.018	-2.469	0.0136
HLA A*24:02	13+	0.883	0.021	-5.844	5.1E-09
DR3	Control	0.950	0.018	-2.815	0.00487
DR3	2-7	0.970	0.020	-1.548	0.122

DR3	7-13	0.962	0.019	-2.006	0.0448
DR3	13+	0.967	0.023	-1.474	0.14
DR4	Control	0.966	0.017	-2.044	0.041
DR4	2-7	1.092	0.019	4.635	3.57E-06
DR4	7-13	1.109	0.018	5.601	2.13E-08
DR4	13+	1.145	0.022	6.208	5.38E-10
DR3/DR4	Control	0.658	0.012	-33.593	<2.2E-16
DR3/DR4	2-7	0.910	0.014	-6.865	6.63E-12
DR3/DR4	7-13	0.870	0.013	-10.386	<2.2E-16
DR3/DR4	13+	0.829	0.016	-11.817	<2.2E-16
DRB1*15:01-DQA1*01:02-DQB1*06:02	Control	1.279	0.017	14.406	<2.2E-16
DRB1*15:01-DQA1*01:02-DQB1*06:02	2-7	0.989	0.019	-0.580	0.562
DRB1*15:01-DQA1*01:02-DQB1*06:02	7-13	0.991	0.018	-0.466	0.641
DRB1*15:01-DQA1*01:02-DQB1*06:02	13+	0.996	0.022	-0.176	0.861
DRB1*07:01-DQA1*02:01-DQB1*03:03	Control	1.086	0.010	8.442	<2.2E-16
DRB1*07:01-DQA1*02:01-DQB1*03:03	2-7	1.009	0.011	0.838	0.402

DRB1*07:01-DQA1*02:01-DQB1*03:03	7-13	1.011	0.011	0.999	0.318
DRB1*07:01-DQA1*02:01-DQB1*03:03	13+	1.017	0.013	1.350	0.177
DRB1*13:02-DQA1*01:02-DQB1*06:09	Control	1.017	0.005	3.230	0.00124
DRB1*13:02-DQA1*01:02-DQB1*06:09	2-7	0.998	0.006	-0.300	0.765
DRB1*13:02-DQA1*01:02-DQB1*06:09	7-13	0.998	0.006	-0.354	0.723
DRB1*13:02-DQA1*01:02-DQB1*06:09	13+	1.004	0.007	0.645	0.519
DRB1*13:01-DQA1*01:03-DQB1*06:03	Control	1.081	0.011	6.872	6.31E-12
DRB1*13:01-DQA1*01:03-DQB1*06:03	2-7	1.007	0.013	0.583	0.56
DRB1*13:01-DQA1*01:03-DQB1*06:03	7-13	1.012	0.012	0.954	0.34
DRB1*13:01-DQA1*01:03-DQB1*06:03	13+	1.013	0.014	0.915	0.36
DRB1*08:01-DQA1*04:01-DQB1*04:02	Control	0.988	0.008	-1.405	0.16
DRB1*08:01-DQA1*04:01-DQB1*04:02	2-7	1.002	0.009	0.222	0.825

DRB1*08:01-DQA1*04:01-DQB1*04:02	7-13	0.993	0.009	-0.817	0.414
DRB1*08:01-DQA1*04:01-DQB1*04:02	13+	0.992	0.011	-0.704	0.481
HLA A*02:01	Control	0.953	0.027	-1.777	0.0756
HLA A*02:01	2-7	1.023	0.030	0.764	0.445
HLA A*02:01	7-13	1.025	0.029	0.852	0.394
HLA A*02:01	13+	1.028	0.035	0.789	0.43
HLA A*02:05	Control	0.990	0.006	-1.731	0.0834
HLA A*02:05	2-7	1.000	0.007	-0.004	0.996
HLA A*02:05	7-13	1.000	0.007	-0.019	0.985
HLA A*02:05	13+	0.989	0.008	-1.416	0.157
HLA A*11:01	Control	1.055	0.013	4.007	6.15E-05
HLA A*11:01	2-7	1.002	0.015	0.109	0.913
HLA A*11:01	7-13	1.016	0.014	1.080	0.28
HLA A*11:01	13+	1.003	0.017	0.163	0.87
HLA B*18:01	Control	0.945	0.013	-4.555	5.24E-06
HLA B*18:01	2-7	1.006	0.014	0.402	0.688
HLA B*18:01	7-13	1.006	0.013	0.413	0.68
HLA B*18:01	13+	0.983	0.016	-1.082	0.279
HLA B*39:06	Control	0.911	0.007	-13.374	<2.2E-16

HLA B*39:06	2-7	0.974	0.008	-3.334	0.000855
HLA B*39:06	7-13	0.945	0.008	-7.531	5.04E-14
HLA B*39:06	13+	0.928	0.009	-8.306	<2.2E-16
HLA B*44:03	Control	1.078	0.013	5.826	5.68E-09
HLA B*44:03	2-7	1.011	0.014	0.732	0.464
HLA B*44:03	7-13	1.009	0.014	0.652	0.514
HLA B*44:03	13+	1.025	0.017	1.468	0.142
HLA B*45:01	Control	1.001	0.005	0.239	0.811
HLA B*45:01	2-7	0.992	0.005	-1.497	0.134
HLA B*45:01	7-13	0.996	0.005	-0.823	0.411
HLA B*45:01	13+	1.004	0.006	0.593	0.553
HLA DPB1*03:01	Control	0.906	0.019	-5.177	2.26E-07
HLA DPB1*03:01	2-7	1.001	0.021	0.054	0.957
HLA DPB1*03:01	7-13	0.983	0.021	-0.847	0.397
HLA DPB1*03:01	13+	0.958	0.024	-1.747	0.0806
HLA DPB1*04:02	Control	1.086	0.018	4.718	2.38E-06
HLA DPB1*04:02	2-7	0.984	0.019	-0.848	0.397
HLA DPB1*04:02	7-13	0.984	0.019	-0.871	0.384
HLA DPB1*04:02	13+	1.004	0.022	0.183	0.855
HLA DPB1*15:01	Control	0.999	0.005	-0.254	0.8

HLA DPB1*15:01	2-7	1.009	0.006	1.541	0.123
HLA DPB1*15:01	7-13	1.009	0.006	1.563	0.118
HLA DPB1*15:01	13+	1.007	0.007	0.978	0.328
