# Supplementary material for: Genetic risk in extremely early onset type 1 diabetes
Source: medRxiv. 2025 Dec 19:2025.12.18.25342362. Preprint. [Version 1] doi: 10.64898/2025.12.18.25342362 (PMC12723774; doi:10.64898/2025.12.18.25342362)
Supplement: Supplement 6 [file media-6.pdf]

Supplementary Table 5. Regression output comparing HLA contribution to Control group including interaction terms.

Predictor	<2 OR	<2 SE	<2 *P*-value	2-7 OR	2-7 SE	2-7 *P*-value	7-13 OR	7-13 SE	7-13 *P*-value	13+ OR	13+ SE	13+ *P*-value
A*24:02	1.06	0.39	8.85E-01	2.27	0.14	2.73E-09	1.41	0.12	3.45E-03	0.81	0.26	4.20E-01
A*24:02 × B*39:06	1.62	0.33	1.39E-01	0.97	0.22	8.73E-01	0.98	0.22	9.12E-01	0.95	0.40	9.00E-01
A*24:02 × DR3-DQ2/DR3-DQ2	1.84	0.52	2.43E-01	1.32	0.35	4.24E-01	1.75	0.34	1.00E-01	0.26	1.08	2.16E-01
A*24:02 × DR3-DQ2/DR4-DQ8	1.25	0.44	6.03E-01	0.66	0.22	6.54E-02	0.85	0.21	4.53E-01	0.84	0.38	6.43E-01
A*24:02 × DR3-DQ2/X	1.90	0.45	1.57E-01	1.09	0.19	6.59E-01	1.74	0.17	1.12E-03	1.49	0.36	2.66E-01
A*24:02 × DR4-DQ8/DR4-DQ8	1.05	0.77	9.53E-01	0.64	0.34	1.88E-01	0.92	0.33	7.88E-01	0.79	0.53	6.51E-01
A*24:02 × DR4-DQ8/X	1.72	0.44	2.18E-01	0.46	0.18	2.58E-05	0.80	0.16	1.61E-01	1.12	0.31	7.15E-01

B*39:06	9.83	0.51	6.97E-06	12.31	0.24	<2.2E-16	6.92	0.23	<2.2E-16	7.14	0.37	1.59E-07
B*39:06 × DR3-DQ2/DR3-DQ2	1.06e+59	0.59	<2.2E-16	4.85e+57	0.73	<2.2E-16	6.16e+58	0.48	<2.2E-16	9.61E-27	9.16E-86	<2.2E-16
B*39:06 × DR3-DQ2/DR4-DQ8	0.17	0.65	6.51E-03	0.12	0.49	1.22E-05	0.17	0.49	4.22E-04	0.05	1.14	9.03E-03
B*39:06 × DR3-DQ2/X	0.72	0.59	5.75E-01	0.52	0.31	4.05E-02	0.48	0.32	2.21E-02	0.25	0.64	3.13E-02
B*39:06 × DR4-DQ8/DR4-DQ8	3.82E-59	NA	NA	0.06	0.69	2.91E-05	0.04	0.79	7.04E-05	0.08	1.12	2.64E-02
B*39:06 × DR4-DQ8/X	0.81	0.55	7.07E-01	0.46	0.30	1.04E-02	0.41	0.30	3.64E-03	0.37	0.50	4.62E-02
DR3-DQ2/DR3-DQ2	64.21	0.24	<2.2E-16	33.88	0.14	<2.2E-16	18.57	0.12	<2.2E-16	18.71	0.17	<2.2E-16
DR3-DQ2/DR4-DQ8	141.56	0.21	<2.2E-16	88.77	0.11	<2.2E-16	46.37	0.09	<2.2E-16	35.04	0.14	<2.2E-16
DR3-DQ2/X	6.64	0.23	<2.2E-16	5.22	0.11	<2.2E-16	3.07	0.08	<2.2E-16	3.07	0.14	2.22E-16
DR4-DQ8/DR4-DQ8	31.95	0.36	<2.2E-16	49.41	0.17	<2.2E-16	26.93	0.15	<2.2E-16	30.17	0.20	<2.2E-16

DR4-DQ8/X	7.67	0.23	<2.2E-16	10.84	0.11	<2.2E-16	7.06	0.08	<2.2E-16	6.65	0.13	<2.2E-16
