# Supplementary material for: Genetic risk in extremely early onset type 1 diabetes
Source: medRxiv. 2025 Dec 19:2025.12.18.25342362. Preprint. [Version 1] doi: 10.64898/2025.12.18.25342362 (PMC12723774; doi:10.64898/2025.12.18.25342362)
Supplement: Supplement 7 [file media-7.pdf]

Supplementary Table 6. Discriminative Performance of T1D-GRS Across Population Centile Thresholds type 1 diabetes diagnosed <2 years in EXE-T1D/EXTEND/PRB cohort and T1DGC cohort combined. Risk of type 1 diabetes diagnosed <2 years calculated using a 0.00012% population prevalence (26). Population centile calculated from UK Biobank European population.

Population Centile	T1D Centile	T1D-GRS	Sensitivity (%)	Specificity (%)	1-Specificity (%)	Youden index	T1D risk (%)
50	1.9	10.159	98.1	44.1	55.9	0.422	0.02
75	3.4	11.815	96.6	69.0	31.0	0.657	0.04
80	3.7	12.185	96.3	74.3	25.7	0.706	0.04
85	5.7	12.599	94.3	79.9	20.1	0.742	0.06
90	8.9	13.115	91.1	86.0	14.0	0.771	0.08
95	20.5	13.882	79.5	92.3	7.7	0.718	0.12
99	51	15.303	49.0	98.0	2.0	0.470	0.29
100	100	19.103	0	100	0	0	NA
