# Supplementary material for: Genetic risk in extremely early onset type 1 diabetes
Source: medRxiv. 2025 Dec 19:2025.12.18.25342362. Preprint. [Version 1] doi: 10.64898/2025.12.18.25342362 (PMC12723774; doi:10.64898/2025.12.18.25342362)
Supplement: Supplement 8 [file media-8.pdf]

Supplementary Table 7. Discriminative Performance of T1D-GRS across Population Centile Thresholds for type 1 diabetes diagnosed (T1D) <2 years in A) EXE-T1D/EXTEND/PRB cohort. Population centile calculated from UK Biobank European population.

Population Centile	T1D Centile	T1D-GRS	Sensitivity (%)	Specificity (%)	1-Specificity (%)	Youden index
50	3.7	10.159	96.3	42.9	57.1	0.392
75	5.8	11.815	94.2	67.6	32.4	0.618
80	6.2	12.185	93.8	72.8	27.2	0.666
85	9.1	12.599	90.9	78.8	21.2	0.697
90	12.0	13.115	88.0	85.1	14.9	0.731
95	25.6	13.882	74.4	91.7	8.3	0.661
99	55.8	15.303	44.2	98.0	2.0	0.422
100	100	19.103	0	100	0	0
