# Supplementary material for: Genetic risk in extremely early onset type 1 diabetes
Source: medRxiv. 2025 Dec 19:2025.12.18.25342362. Preprint. [Version 1] doi: 10.64898/2025.12.18.25342362 (PMC12723774; doi:10.64898/2025.12.18.25342362)
Supplement: Supplement 9 [file media-9.pdf]

Supplementary Table 8. Discriminative Performance of T1D-GRS across Population Centile Thresholds for type 1 diabetes diagnosed (T1D) <2 years in T1DGC cohort. Population centile calculated from UK Biobank European population.

Population Centile	T1D Centile	T1D-GRS	Specificity (%)	Sensitivity (%)	1-Specificity (%)	Youden index
50	0.6	10.159	99.4	45.2	54.8	0.447
75	1.7	11.815	98.3	70.6	29.4	0.689
80	2.0	12.185	98.0	75.9	24.1	0.739
85	3.4	12.599	96.6	81.2	18.8	0.778
90	6.8	13.115	93.2	87.0	13.0	0.802
95	17.0	13.882	83.0	93.0	7.0	0.759
99	47.7	15.303	52.3	98.0	2.0	0.503
100	100	19.103	0	100	0	0
