# Supplementary material for: Genetic risk in extremely early onset type 1 diabetes
Source: medRxiv. 2025 Dec 19:2025.12.18.25342362. Preprint. [Version 1] doi: 10.64898/2025.12.18.25342362 (PMC12723774; doi:10.64898/2025.12.18.25342362)
Supplement: Supplement 10 [file media-10.pdf]

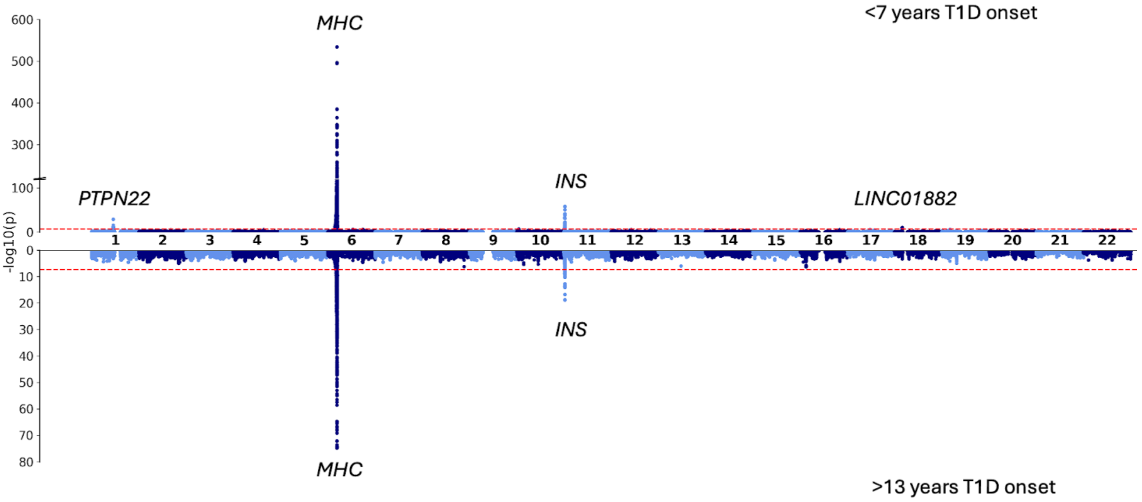

Supplementary Figure 1. Miami plot for variants associated with <7 years type 1 diabetes (T1D) onset (top) and >13 years T1D onset (bottom). Red line represents *P*=5x10^{-8}. Loci are labelled based on nearest gene.
