# Supplementary material for: Genetic risk in extremely early onset type 1 diabetes
Source: medRxiv. 2025 Dec 19:2025.12.18.25342362. Preprint. [Version 1] doi: 10.64898/2025.12.18.25342362 (PMC12723774; doi:10.64898/2025.12.18.25342362)
Supplement: Supplement 11 [file media-11.pdf]

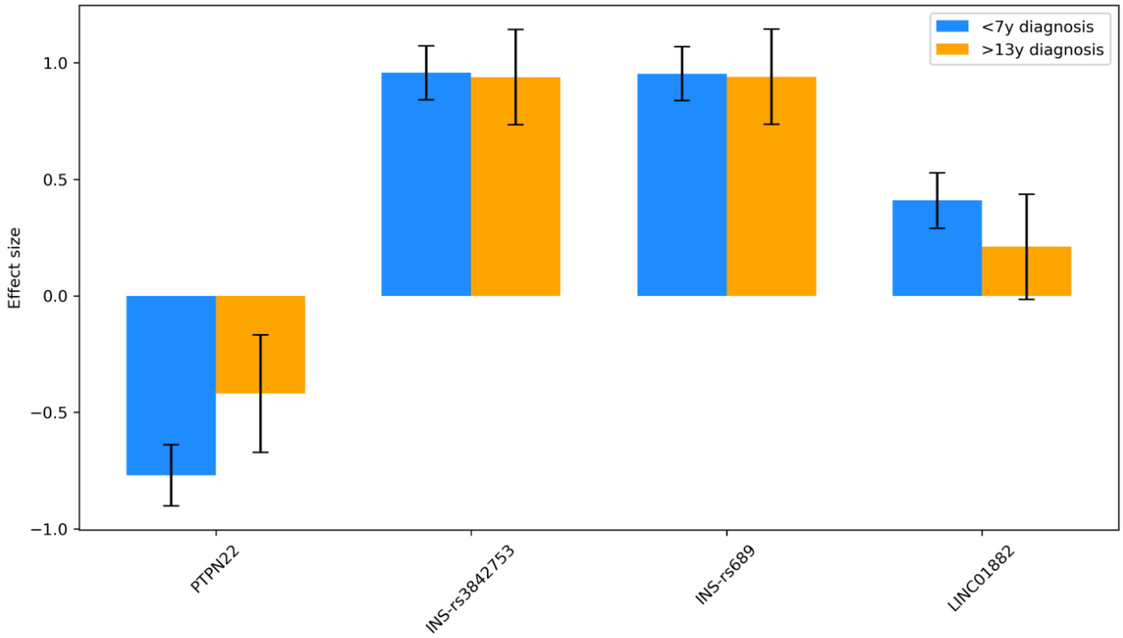

Supplementary Figure 2. Effect sizes of variants significant in association testing for <7 years (blue) and >13 years (orange) type 1 diabetes onset. Error bars represent 95% confidence intervals.
