# Supplementary material for: Genetic risk in extremely early onset type 1 diabetes
Source: medRxiv. 2025 Dec 19:2025.12.18.25342362. Preprint. [Version 1] doi: 10.64898/2025.12.18.25342362 (PMC12723774; doi:10.64898/2025.12.18.25342362)
Supplement: Supplement 12 [file media-12.pdf]

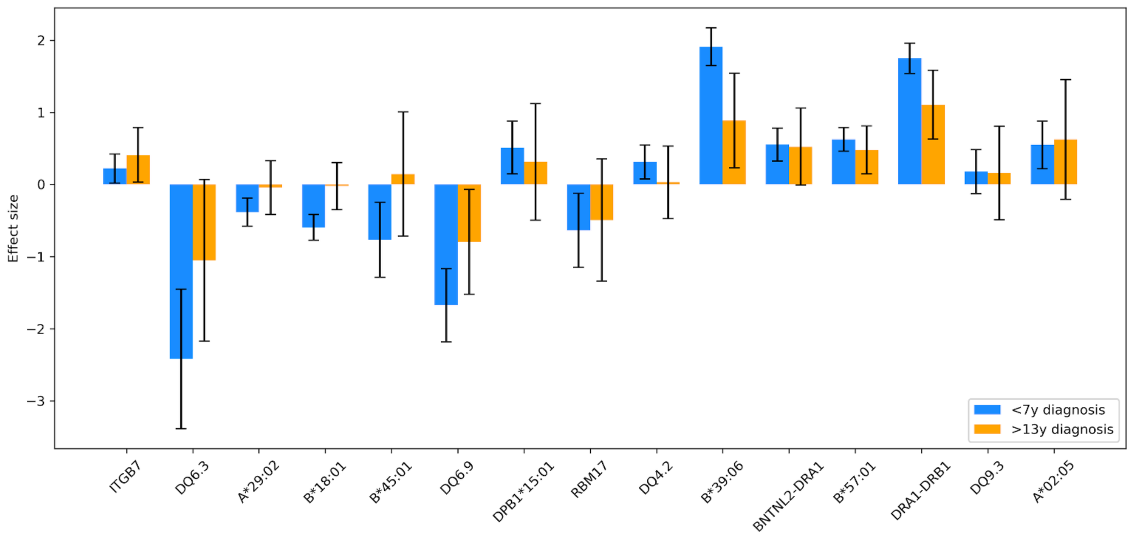

Supplementary Figure 3. Effect sizes for nominally significant (P<0.05) T1D-GRS for <7 years (blue) and >13 years (orange) type 1 diabetes onset. *= P<0.05. Error bars represent 95% confidence intervals.
