# Supplementary material for: Genetic risk in extremely early onset type 1 diabetes
Source: medRxiv. 2025 Dec 19:2025.12.18.25342362. Preprint. [Version 1] doi: 10.64898/2025.12.18.25342362 (PMC12723774; doi:10.64898/2025.12.18.25342362)
Supplement: Supplement 13 [file media-13.pdf]

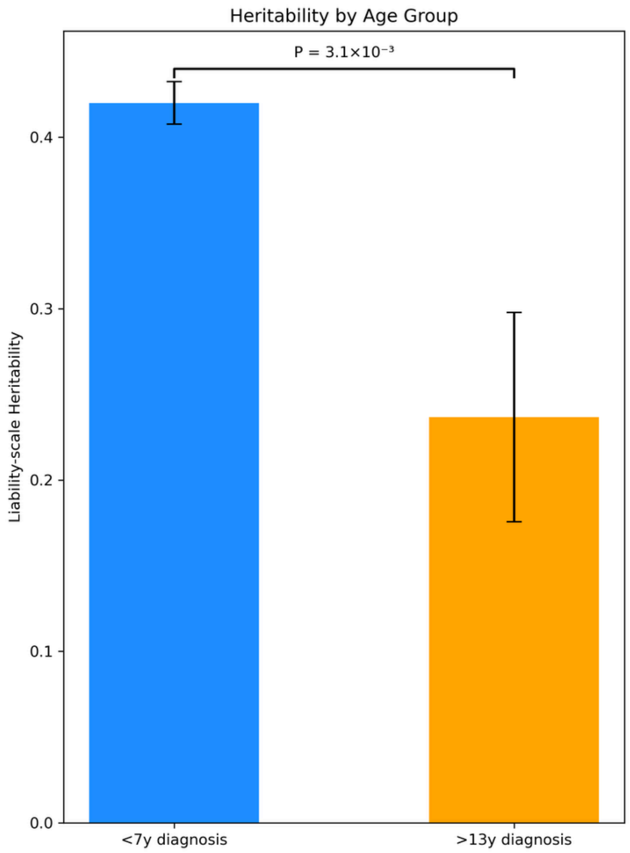

Supplementary Figure 4. Liability-scale heritability of <7 years onset (blue) and >13 years onset (orange). Error bars represent standard error.
