# Supplementary material for: Genetic risk in extremely early onset type 1 diabetes
Source: medRxiv. 2025 Dec 19:2025.12.18.25342362. Preprint. [Version 1] doi: 10.64898/2025.12.18.25342362 (PMC12723774; doi:10.64898/2025.12.18.25342362)
Supplement: Supplement 15 [file media-15.pdf]

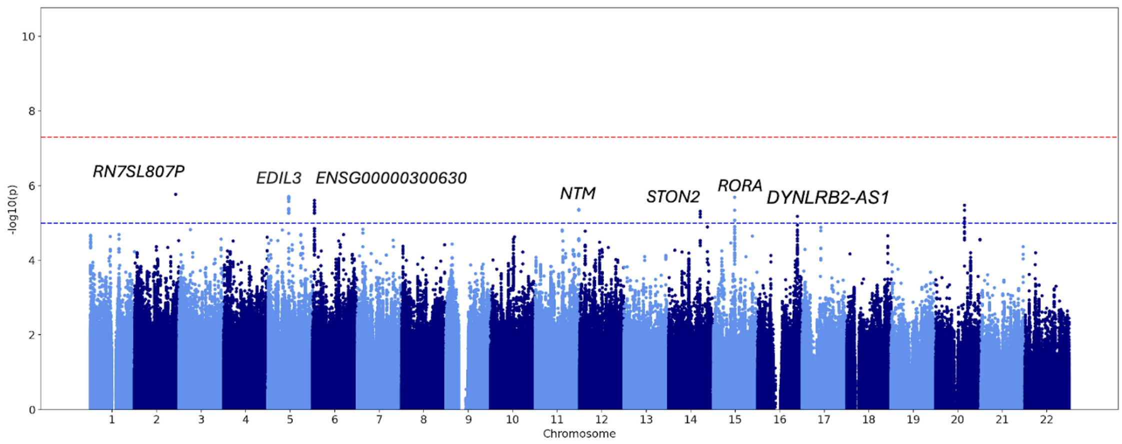

Supplementary Figure 6. Manhattan plots from meta-analysis of continuous phenotype age of diagnosis for EXET1D/EXTEND/PRB cohorts and T1DGC cohort. Red line represents P=5x10^{-8}, blue line represents p=1x10^{-5}. Loci are labelled based on nearest gene.
